# Radiopharmacy services in South African academic hospitals: implications for planning radiopharmacy services in Africa

**DOI:** 10.3389/fnume.2026.1822893

**Published:** 2026-04-29

**Authors:** David Kimani, Beverley Summers, Lerato Mosima

**Affiliations:** Department of Pharmaceutical Sciences, School of Pharmacy, Sefako Makgatho Health Sciences University, Pretoria, South Africa

**Keywords:** nuclear medicine, PET-CT, radiopharmaceuticals, radiopharmacy, SPECT-CT, staffing norms

## Abstract

**Background:**

Nuclear medicine (NM) is being embraced by undeveloped countries as a specialty. Due to the high costs involved in establishing new NM and radiopharmacy centres, proper planning is essential. This study describes the pattern of radiopharmacy services and staffing in NM departments in South African Academic hospitals (SAAHs); which can aid planning of new NM and radiopharmacy centres in similar settings.

**Methods:**

The study was retrospective, descriptive, and quantitative. An anonymized questionnaire was sent via Survey Monkey® to the nine SAAHs to determine nuclear medicine patient numbers by scan type, identify cameras, equipment, staffing levels, commonly used radiopharmaceuticals, and define the basic requirements for establishing new facilities.

**Results:**

The response rate was 78% (7/9 hospitals). Average number of NM patients seen per hospital was 7,040 for 2022. All hospitals had SPECT-CT cameras; six had PET-CT cameras and one referred PET patients to a private hospital. All hospitals had NM physicians (range 2–13, average 5) and at least one medical physicist (range 1–4). Only two hospitals had radiopharmacists (only one in each).

**Conclusion:**

To promote optimal patient services and support research in SAAHs, norms must be developed that are applicable to Africa. Appropriate staff quotas and equipment are required for the African setting to promote optimal use of radiopharmaceuticals and enable NMs to operate to their full potential. Key things to help in planning and establishing new radiopharmacy centers include; adopting centralized radiopharmaceutical production and aligning staffing with international guidelines.

## 1 Introduction

Nuclear medicine plays a critical role in imaging of the human body's internal organ systems to provide visual representations of metabolic processes in specific organs (1). It utilises small amounts of radiopharmaceutical products which are vectors tagged to radioactive atoms. These radiopharmaceuticals are administered in the body for diagnosis and treatment of serious medical conditions (1). Diagnostic radiopharmaceuticals, once administered in the body, localize in target organs and emit radiation which is captured on external detectors, enabling imaging of the disease extent in the body. Molecular imaging enables medical practitioners to make appropriate and personalized decisions for each and every patient (2). Therapeutic radiopharmaceuticals once in the body are taken up by target cells and emit radiation that leads to the death of the targeted cells (3).

Globally, nuclear medicine plays an important role in the patient management matrix. Nuclear medicine services uptake has increased over time in many developed countries (4). In Africa, there have also been developments made in nuclear medicine. In 2022 there were reported to be 17 planar-only gamma cameras, 132 gamma cameras, 87 SPECT gamma cameras, 28 SPECT-CT gamma cameras, 11 PET scanners and 6 cyclotrons in Africa (5). South Africa and North African countries have similar development patterns to some developed countries while other African countries have lagged far behind (4).

South Africa (SA) has an established infrastructure for the production of radionuclides (6). SA offers post-graduate training in nuclear medicine and radiopharmacy and has partnered with the International Atomic Energy Agency (IAEA) to provide regional training courses which support African countries.

An audit of patient statistics in just two South African academic hospitals in Johannesburg from January 2004 to December 2019 showed that a total of 120,033 nuclear medicine procedures were performed over the period (7). This number shows that there is demand for nuclear medicine and radiopharmacy services which can be attributed to the shift from communicable to non-communicable diseases and new disease outbreaks bringing to the fore the need to strengthen health systems especially with regards to nuclear medicine which plays a vital role in the diagnosis and treatment of some of these diseases (8). This increased need will require further research to develop new radiopharmaceuticals, establish nuclear medicine and radiopharmacy centres and train staff in these areas. There will also be a need for health care managers to have important information which can be determined from service uptake trends in already -existing nuclear and radiopharmacy centres which they can use for planning of new nuclear medicine and radiopharmacy services and predicting future needs for expansion of existing facilities.

The high costs of nuclear medicine infrastructure, radiopharmaceuticals and specialist staff dictate that proper planning be done before establishing these centres, so that services may be tailored towards needs and be sustainable, especially in low-resource settings. It is in this regard, that an analysis of radiopharmacy services at South African academic hospitals was carried out to give updated data on the types of scans that are carried out, the commonly used radiopharmaceuticals, the staffing levels and the patient statistics. The findings were used to define the basic requirements needed for the establishment of new radiopharmacy centres in similar settings.

## 2 Materials and methods

The study was retrospective, descriptive, and quantitative. It involved sending out anonymized questionnaires to heads of department in the nine South African Academic Hospitals for the year 2022. The questionnaire was generated through Survey Monkey®. A preliminary statement explained that continuation with the survey indicated consent. The questionnaire collected information regarding the hospital bed capacity, the total outpatient statistics, nuclear medicine patient statistics against scan types, staffing levels and needs, and the availability of different types of equipment.

The data collected was provided by the respective nuclear medicine departments and since it was obtained from their databases, it was deemed reliable. The data shows a true reflection of patient statistics for the departments and utilization of radiopharmaceutical services since the nuclear medicine and radiopharmacy centers recorded their patient data. Consistency was achieved by the use of a similar data collection form across the identified hospitals. The use of 2022 data enhanced validity while the data collection form had been tailored to include IAEA parameters that included radiopharmaceuticals used, equipment and facilities present and types of scans. This also ensured that errors were minimized and that only necessary data was captured.

There are different types of bias and the main potential types of bias in this study were addressed as follows;

- Selection bias did not arise as the researcher included all academic hospitals with Nuclear Medicine Departments in South Africa.
- Interviewer bias did not arise as no interviews were conducted.
- Recall bias was minimised by concentrating only on data for the year 2022.
- Acquiescence bias did not occur as there were no questions that elicited a favourable answer as binary responses were not part of the questions in the questionnaire.
- Social desirability bias was also eliminated as respondents were anonymised.
- Non-response bias may have arisen even though the researcher kept the questionnaire as simple as possible with minimal questions which had clear wording and instruction.

An analysis of patient statistics data was performed by categorizing scans as either SPECT, PET or as radionuclide therapy. The most frequently used radiopharmaceuticals were identified. A comparison of the patient statistics in terms of numbers of nuclear medicine scans performed in the year 2022 was done. Staff to patient scan ratios comparisons were also made.

The number of each cadre of staff working in the respective nuclear medicine departments was also determined. Data obtained was compared with the IAEA staff modelling recommendations which also relates to the IAEA Operational levels. The comparison was to determine whether the IAEA staff modelling levels were achievable for new radiopharmacy centers (5).

## 3 Results

The results obtained from the seven responding academic hospitals are grouped as follows:

- Nuclear medicine patient numbers by scan type for the year 2022.
- Radiopharmaceuticals used.
- Cameras and equipment in the various nuclear medicine departments.
- Staffing levels for the various nuclear medicine departments.
- Additional staff stated to be required in each hospital.

The response rate was 78% (7/9) hospitals. Over the nine hospitals, bed numbers ranged from 450 to 3,400 beds. Of the seven responding hospitals, bed numbers ranged from 846 to 1,650 beds. Only two hospitals reported hospital outpatient numbers for 2022 (427,465 and 107,478 respectively). In two of the responding hospitals, the ratio of in-patients to outpatients seen in the nuclear medicine department was 1:6 and 1:17. This is a clear indication that the majority of patients seen in the respective nuclear medicine departments were outpatients.

### 3.1 Overview of nuclear medicine patient numbers by scan type for the year 2022

**Figure 1:**
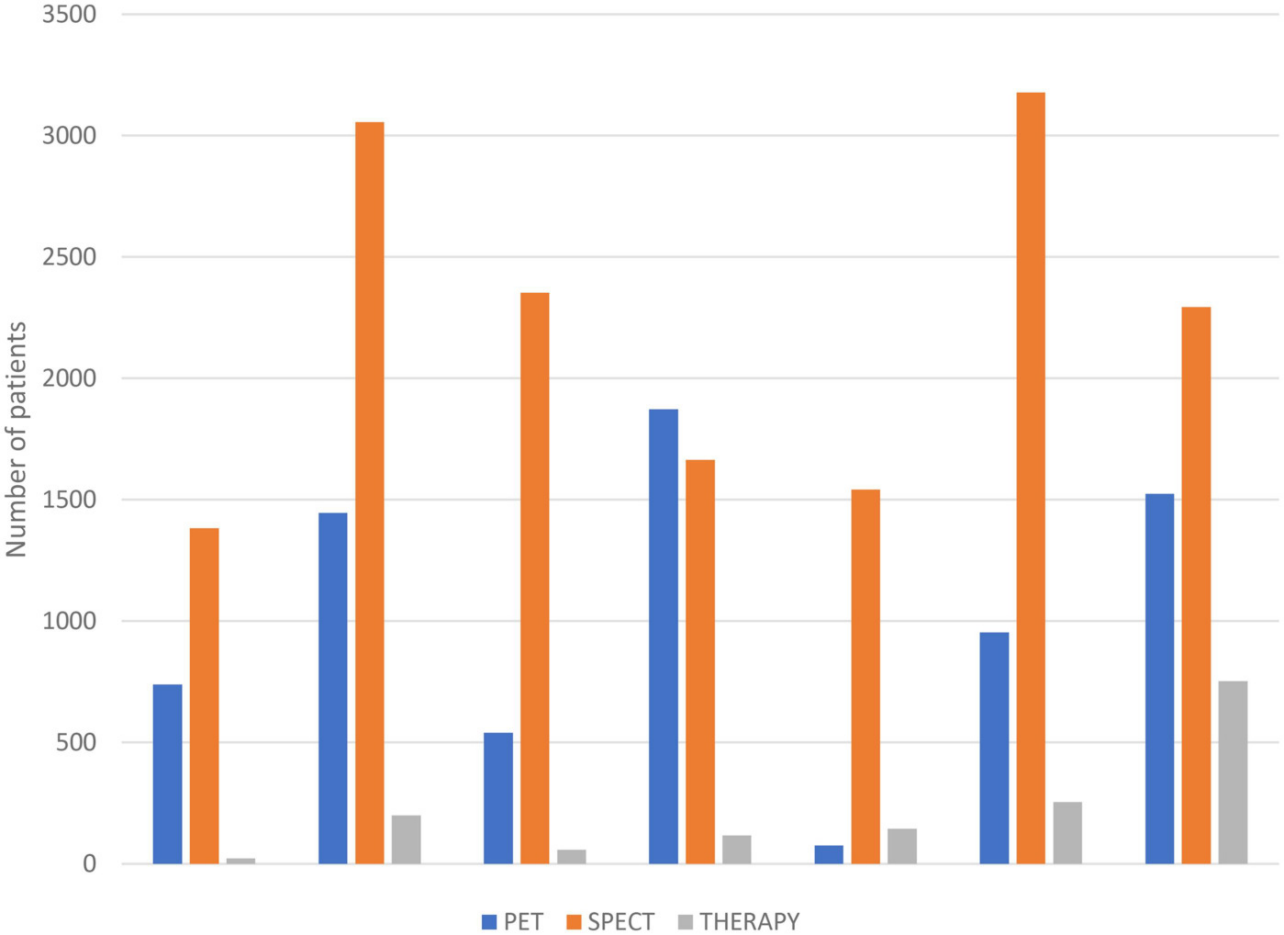
Patient numbers for PET, SPECT and radionuclide therapy for the seven SA academic hospitals, 2022.

All seven hospitals carried out PET and SPECT scans and radionuclide therapy in the year 2022. The total nuclear medicine patients seen was 24,164 (mean per hospital 3,452 ± 1,103.8 SD. Patient numbers across the responding hospitals ranged between 1,762 and 4,702. In summary, Figure 1 shows the comparison of patient statistics against PET, SPECT and radionuclide therapy carried out in 2022 in South African academic hospitals that participated in this study.

#### 3.1.1 PET scans and radiopharmaceuticals

In the seven hospitals, a total of 7,150 patients underwent PET scans in the year 2022 (average 1,021 patients per hospital +/- 581.6 SD). [^18^F] F-FDG was the highest used positron emitter with 5,247 patients (average750 +/- 377.8 SD) with patient number ranges of 76 to 1,226 across the responding hospitals followed by [^68^Ga] Ga-PSMA with a total of 1,236 patients (average 177 +/- 190.3 SD) with patient ranges of 27 to 600.

**Figure 2:**
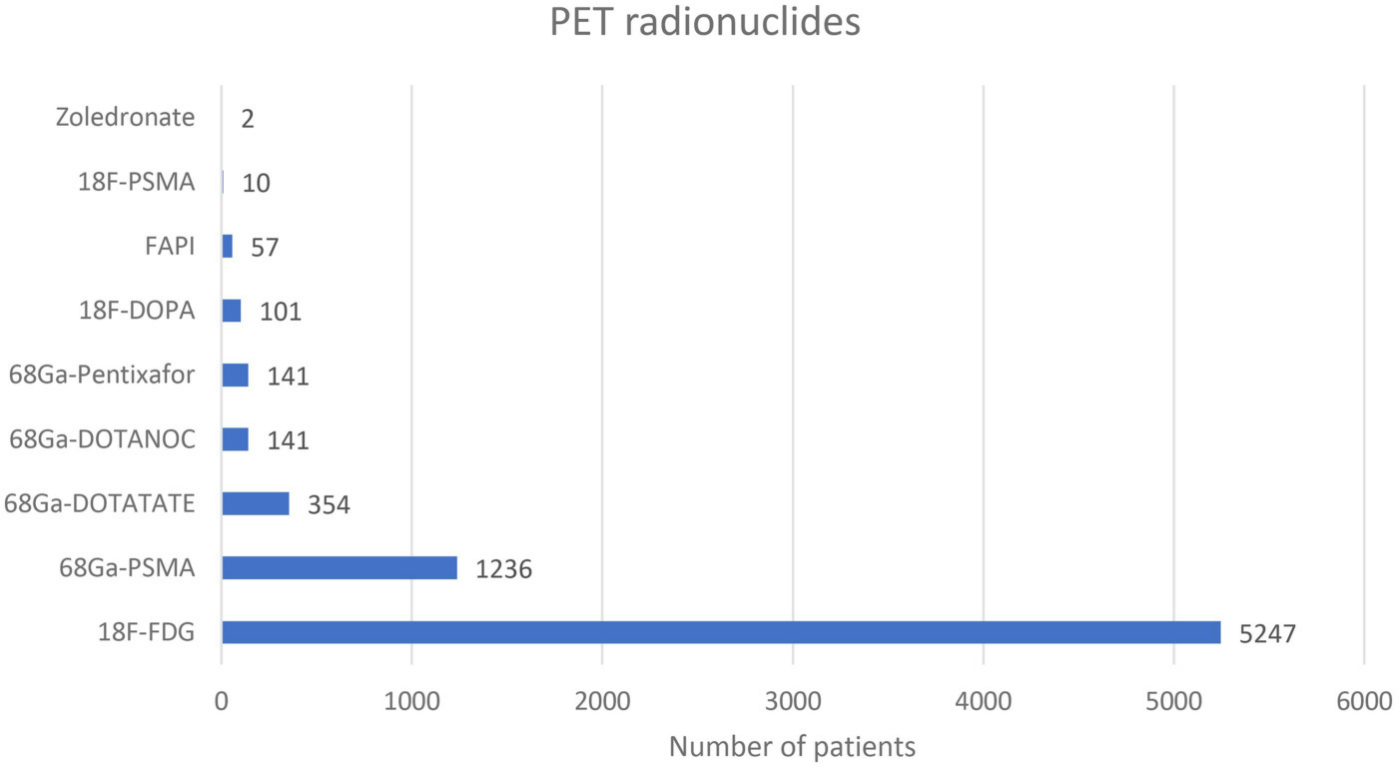
PET scans vs. patient numbers for the year 2022.

Figure 2 shows the total number of patients who underwent PET scans in the seven hospitals in the year 2022.

#### 3.1.2 SPECT scans and radiopharmaceuticals

SPECT scans were the most common nuclear medicine scan type in the year 2022 with a total of 15,453 patients scanned in the academic hospitals. All hospitals carried out SPECT scans with an average of 2,208 patients per hospital (+/- 666.2 SD) with the number of patients ranging from 1,382 to 3,178 across the hospitals. Bone scans using the radiopharmaceutical Technetium-99 m tagged to Methylene Di-Phosphonate (MDP) had the highest number of SPECT patients at 3,540 (average 506+/−170.7 SD, range 324 to 782). It was followed by ventilation and perfusion lung scan which had 2,512 patients (average 359 +/- 190.4 SD, range of 46 to 596).

Table 1 shows the number of patients (by hospital and radiopharmaceutical) who underwent SPECT scans in the seven hospitals in the year 2022.

**Table 1:** SPECT scans vs. patient numbers for the year 2022.

Type of scan	Number of SPECT patients (2022)							
Bone MIBI	2	0	1	0	0	0	0	**3**
Bone MDP	747	451	348	324	465	782	423	**3,540**
Thyroid scan	139	321	144	99	188	265	330	**1,486**
Iodine-123	19	21	32	38	2	0	15	**127**
Renal DTPA	43	6	0	7	0	0	0	**56**
Renal MAG-3	0	330	249	0	0	195	84	**858**
Parathyroid scan	0	55	62	83	17	73	44	**334**
Lung (V&Q)	152	596	289	46	545	400	484	**2,512**
Renal DMSA	1	8	1	43	0	2	54	**109**
Renal captopril	16	0	0	5	2	0	0	**23**
Heart MIBI	139	258	247	482	132	153	536	**1,947**
Heart MUGA	0	0	0	0	0	1,012	279	**1,291**
WBC labelling	17	19	8	0	4	20	0	**68**
RBC labelling	2	440	684	4	6	1	1	**1,138**
HIDA	0	6	1	28	3	0	2	**40**
MIBG	3	13	0	5	23	26	1	**71**
GIT bleed	1	1	1	426	4	17	7	**457**
Gallium 67	18	0	1	0	0	3	0	**22**
Heart thallium	0	0	0	0	0	4	0	**4**
Merkel's diverticulum	0	2	1	2	4	3	2	**14**
Lymph: sentinel mapping	14	48	18	66	19	65	20	**250**
Lymph: scintigraphy	8	1	45	2	1	4	0	**61**
Milk scan	7	14	4	2	0	0	5	**32**
Testicular	53	0	0	0	0	0	0	**53**
Subclavian	1	0	0	0	0	0	0	**1**
Dacrocintigraphy	0	0	0	0	1	0	0	**1**
Tin colloid	0	3	0	1	0	1	0	**5**
99mTc-PSMA	0	00	106	0	38	0	0	**144**
Bone marrow (nanocolloid)	0	0	1	0	0	0	0	**1**
Gastric emptying	0	3	1	0	4	17	5	**30**
Lu-177 scan	0	49	0	0	0	0	0	**49**
Brain death	0	7	0	0	0	0	0	**7**
R to L shunt	0	3	0	0	0	0	0	**3**
Iodine-131 diagnostic	0	14	11	0	12	102	0	**139**
GFR-DTPA	0	387	76	0	0	14	1	**478**
Lung Q	0	0	2	0	0	11	0	**13**
Lung shunt	0	0	1	0	0	0	0	**1**
Amyloidosis (cardiac)	0	0	0	0	5	0	0	**5**
Peritoneal leakage	0	0	0	0	3	0	0	**3**
Tektrotide	0	0	0	0	13	4	0	**17**
99mTc-ECDG	0	0	0	0	51	0	0	**51**
HMPAO Brain	0	0	5	0	0	2	0	**7**
Thyrogen	0	0	0	0	0	2	0	**2**
Total	**1,382**	**3,056**	**2,339**	**1,663**	**1,542**	**3,178**	**2,293**	**15,453**

Bold values represents Total scans across each row.

Two of the seven responding hospitals reported the use of [^99m^Tc] Tc-PSMA with a total of 144 patients showing that the radiopharmaceutical is beginning to play a vital role in patient management.

Bone MDP and lung V&Q were the most common SPECT scans with 3,540 and 2,512 patients respectively.

The data obtained under SPECT scans is a clear indication of the wide use and application of SPECT imaging modality in renal, skeletal, endocrine, cardiovascular, respiratory, infection & inflammation, gastrointestinal and the lymphatic system. SPECT imaging plays a vital role in any nuclear medicine department.

#### 3.1.3 Radionuclide therapy

All the seven responding hospitals carried out radionuclide therapy. Radionuclide therapy had the lowest number of patients in 2022 compared to PET and SPECT radiopharmaceuticals. A total of 1,561 patients underwent radionuclide therapy in the academic hospitals in 2022 (Average 223+/- 227.4 SD) with the number of patients ranging between 22 and 744.

**Figure 3:**
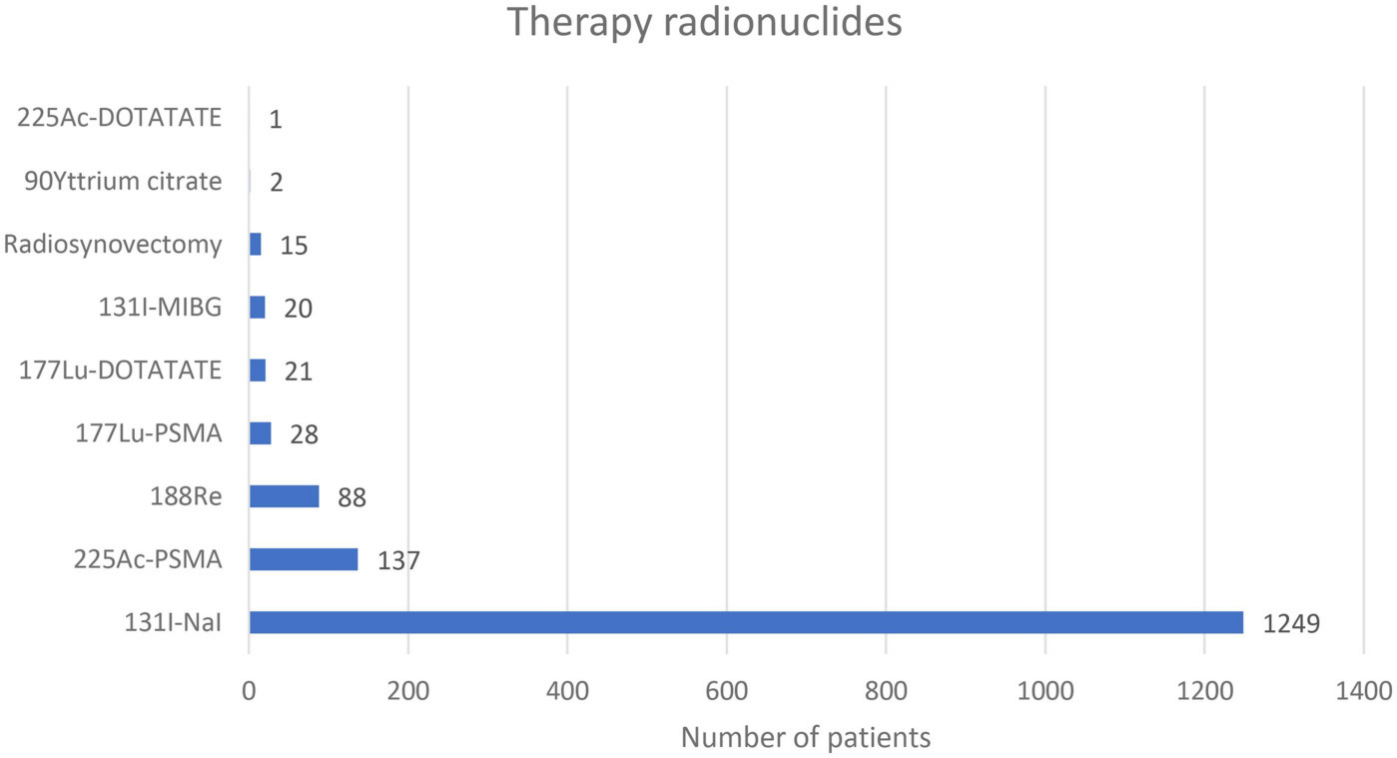
Radionuclide therapy vs. patient numbers for the year 2022.

Figure 3 shows the number of patients who underwent radionuclide therapy in the seven hospitals in the year 2022.

Iodine-131 sodium iodide (1,249 patients, 80%) was the most commonly used radionuclide therapy followed by [225Ac] Ac-PSMA with 137 patients (8.8%) as illustrated in Figure 3.

Iodine-131 treatment for Grave's disease has been the most common nuclear medicine outpatient therapeutic procedure with thyroid cancer treatment being the most common therapeutic inpatient procedure. Lutetium-177, actinium-225, samarium-153 and strontium-89 are available in South Africa as radionuclide therapies (4).

### 3.2 Cameras and equipment

All the participating hospitals had SPECT-CT cameras while only six of the seven responding hospitals had PET-CT cameras. The remaining hospital referred PET patients to a private hospital. The IAEA recommends that nuclear medicine departments should have at least one SPECT-CT Only three hospitals had hot cells and two of these had just one radiopharmacist each. None of the hospitals had an in-house cyclotron. All the hospitals reported having a technetium-99 m generator signifying the important role it plays in SPECT-CT scans.

Radiation protection measures were in place in all the seven hospitals which is evident because of the presence of lead shielding and radiation monitors in the hospitals. All hospitals answered in the affirmative that they were carrying out aseptic admixing despite only three hospitals having clean rooms. The details of equipment, facilities and cameras available is as shown in Table 2.

**Table 2:** Number of cameras and equipment per hospital.

Equipment	Hospitals							Average
Gamma cameras (planar only)	0	2	1	2	1	0	0	0.9
SPECT-CT gamma cameras	1	2	2	1	3	2	2	1.9
PET-CT gamma cameras	1	1	1	1	0	1	1	0.9
Hot-cells	1	2	0	0	0	0	1	0.6
68Ga-Generator	0	1	0	1	0	1	1	0.6
99mTc-Generator	2	2	2	1	1	2	1	1.6
Cell labelling facility	0	1	1	1	1	0	0	0.6
Cyclotron	0	0	0	0	0	0	0	0
Dose calibrators	5	5	5	4	3	2	4	4
Lead shielded laminar airflow	1	4	2	1	1	0	1	1.4
Centrifuge (blood products)	0	3	3	2	1	0	1	1.4
68Gallium synthesis unit	1	1	0	1	0	1	0	0.6
Radiation monitor	7	2	4	1	3	2	4	3.3
Clean room	2	0	1	0	0	0	1	0.6
SPECT gamma camera single head	0	0	0	0	0	0	1	0.1

### 3.3 Staff levels

In this study, all the seven hospitals that responded have nuclear medicine physician numbers ranging from 2 to 13 with an average of 5. None of the hospitals had a cyclotron operator. One hospital had 4 medical physicists while the rest had 1 each. Only two hospitals have a radiopharmacist with one each.

From the data supplied, the calculation of ideal staff levels based on the IAEA International Research Integration System (IRIS) system was difficult. The IRIS-projected number of radiopharmacists for four of the academic hospitals was 3.4, which seems realistic. The number of nuclear medicine physicians for the hospitals ranged from 1.8 to 5.1, which seems inadequate, especially for the teaching setting.

The lack of cyclotron operators in the hospitals can be explained by the fact that private companies invest in cyclotrons and hence supply the cyclotron-produced radioisotopes to the academic hospitals facilities. This situation is evident not only in South Africa but also in Egypt and Morocco (4) and also in Kenya and Nigeria.

The IAEA continues to support African countries in human resource capacity building which has seen countries such as South Africa offer postgraduate training for nuclear physicians whom they also recruit in both public and private nuclear medicine centres (4). The South African Society of Nuclear Medicine (SASNM) reports that it has more than 200 members who include nuclear medicine physicians, radiographers, physicists, radiopharmacists and scientists from various specialities.

Table 3 shows the number of full-time equivalent staff with ratio calculations for each staff category in relation to the PET, SPECT, and radionuclide therapy patients treated at each hospital in the year 2022. This calculation illustrates the number of patients per single staff category member and also indicates IAEA norms for that staff category.

**Table 3:** The number of full-time equivalent staff with ratios to each type of scan.

Staff cadre	Full-time equivalent staff per Academic Hospital for the year 2022							Average
NM physicians (registrars)	**6 (4)**	**4 (4)**	**3 (0)**	**4 (4)**	**2 (0)**	**13 (0)**	**3 (3)**	**5 (2)**
IAEA NORMS	2.8	3.8	2.2	3.5	1.8	4.3	5.1	
PET scans per NM physician	73:1	180:1	180:1	234:1	38:1	73:1	254:1	146:1
SPECT scans per NM physician	138:1	382:1	784:1	208:1	771:1	244:1	382:1	316:1
Therapy per NM physician	2:1	25:1	19:1	14:1	72:1	20:1	125:1	32:1
Total scans per NM physician	214:1	588:1	988:1	457:1	881:1	337:1	762:1	493:1
Medical physicist	**1**	**1**	**1**	**1**	**4**	**1**	**1**	**1**
PET scans/medical physicist	739:1	1,446:1	539:1	1,873:1	19:1	953:1	1,524:1	1,021:1
SPECT scans/medical physicist	1,382:1	3,056:1	2,353:1	1,663:1	386:1	3,178:1	2,293:1	2,210:1
Therapy/medical physicist	22:1	200:1	58:1	117:1	36:1	256:1	752:1	221:1
Total scans/medical physicist	2,143:1	4,702:1	2,964:1	3,653:1	1,762:1	4,385:1	4,569:1	3,454:1
Medical physicist intern	**1**	**0**	**0**	**0**	**0**	**0**	**0**	
Radiopharmacist	**1**	**1**	**0**	**0**	**0**	**0**	**0**	
IAEA NORMS	3.4	3.4				3.4	3.4	
PET scans/radiopharmacist	739:1	1,446:1						
SPECT scans/radiopharmacist	1,382:1	3,056:1						
Therapy/radiopharmacist	22:1	200:1						
Total scans/radiopharmacist	2,143:1	4,702:1						
Radiopharmacist intern	**1**	**0**	**0**	**0**	**0**	**0**	**0**	
Radiation protection officer	0	0	1	0	0	1	1	
PET scans/RPO			539:1			953:1	1,524:1	
SPECT scans/RPO			2,353:1			3,178:1	2,293:1	
Therapy/RPO			58:1			256:1	752:1	
Cyclotron operators	**0**	**0**	**0**	**0**	**0**	**0**	**0**	**0**
NM technologists/radiographers	**5**	**9**	**10**	**9**	**8**	**13**	**8**	**9**
IAEA NORMS	2.2	4.2				2.1	4.0	
PET scans/ NM technologists	148:1	161:1	54:1	208:1	10:1	73:1	191:1	113:1
SPECT scans/ NM technologists	277:1	140:1	235:1	185:1	193:1	244:1	287:1	246:1
Therapy/NM technologists	4:1	22:1	6:1	13:1	18:1	20:1	94:1	25:1
Total scans/NM technologists	429:1	522:1	296:1	406:1	220:1	337:1	571:1	384:1
Nuclear medicine/Radiography students	8	0	0	0	0	12	0	3
Specialist nurses	8	3	1	6	2	3	3	4
IAEA NORMS	1.8	5.5					15.3	
PET scans/specialist nurses	92:1	482:1	539:1	312:1	38:1	318:1	508:1	255:1
SPECT scans/specialist nurses	173:1	1,019:1	2,353:1	277:1	771:1	1,059:1	764:1	552:1
Therapy/specialist nurses	3:1	67:1	58:1	20:1	72:1	85:1	251:1	55:1
Total no. of staff	**35**	**22**	**16**	**24**	**16**	**43**	**19**	**24**

Bold values represents the total number of each staff category.

**Figure 4:**
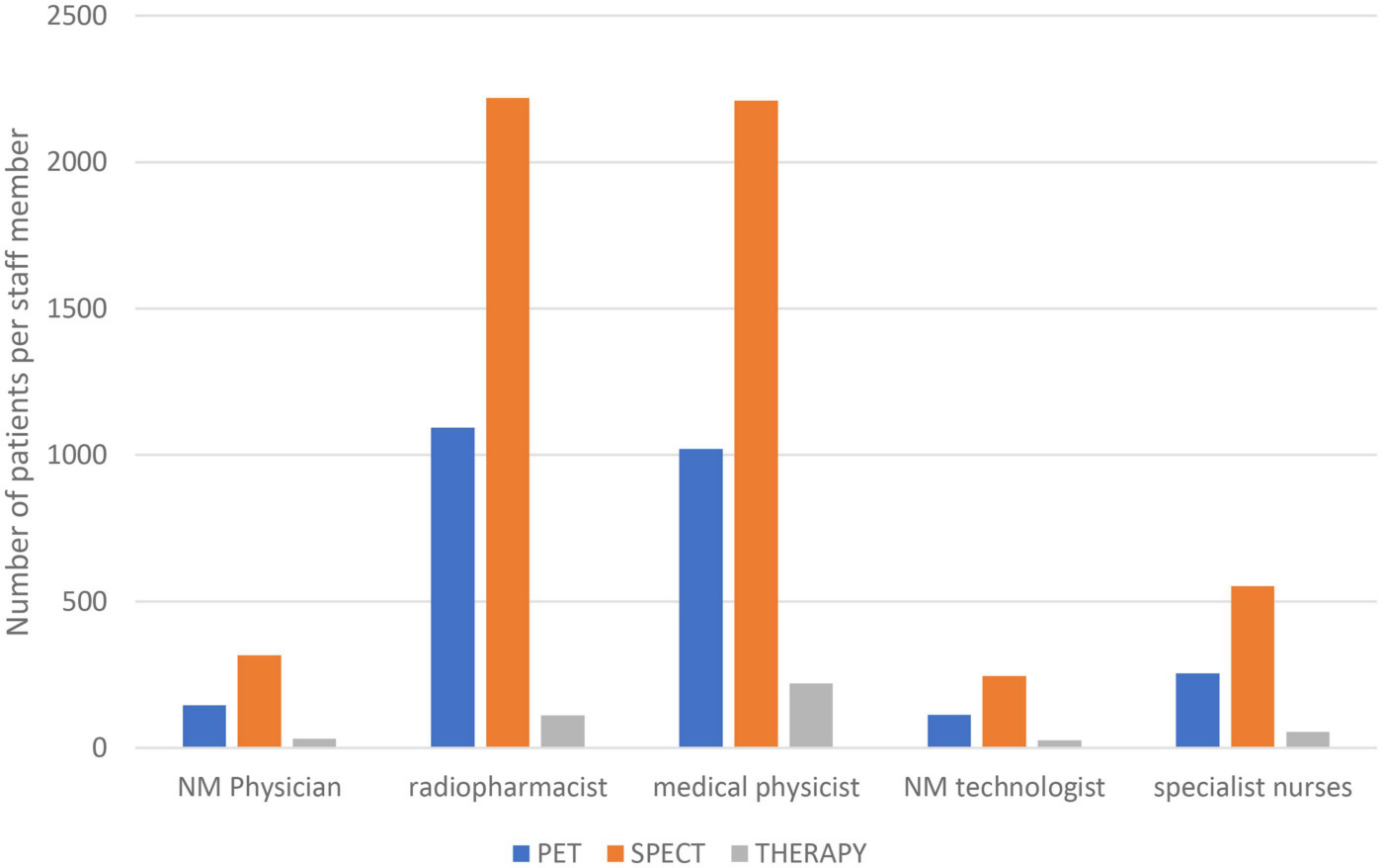
Average patient to key staff ratios for PET, SPECT or therapy for South African academic hospitals, 2022.

A determination of full-time equivalent staff per academic hospital allowed for the comparison of the number of the main staff to the average number of scans conducted in each hospital. On average, over the year, there were 146 PET scans, 316 SPECT scans and 32 radionuclide therapy per one nuclear medicine physician. For the two hospitals with a radiopharmacist each, on average, there were 1,093 PET scans, 2,219 SPECT scans and 111 radionuclide therapy for each radiopharmacist. There were 113 PET, 246 SPECT scans and 25 radionuclide therapy per one nuclear medicine technologist on average. The comparison of key staff to patient numbers per scan is as shown by Figure 4.

### 3.4 Additional staff for the hospitals

All the respondents reported not having enough nuclear medicine/radiopharmacy staff in their respective departments. The main reasons they gave for the requirement of additional staff were;

(a) The bulk of radiopharmacy work falling onto one individual.
(b) Overall growth of the department and increased workload in the department.
(c) Need for an in-house cyclotron and radiopharmacist for in-house production of radiopharmaceuticals.
(d) Increased nursing staff for proper triaging of patients.
(e) Additional coverage during leave cycles.
(f) Opening up time for research.

## 4 Discussion

The findings of this study provide an important highlight of trends in nuclear medicine and radiopharmacy utilisation, workforce capacity and infrastructure across South African academic hospitals while also revealing significant gaps when benchmarked against international standards.

The World Nuclear Association (9) reports that about one person in 50 in the developed countries is subjected to a diagnostic nuclear medicine procedure and over 100,000 hospitals worldwide use radioisotopes in medicine. About 90% are diagnostic, with technetium-99 m being the most common radioisotope accounting for about 80%. It further reports that the use of diagnostic radioisotopes is increasing at over 10% annually. This study data for the SA academic hospitals does not reflect a 10% annual growth. In the 15 years from 2004 to 2019, two academic hospitals performed just over 120,000 scans. On a very rough average that is 4,000 scans a year (7). In this study, the total scans for the year 2022 for the seven hospitals was 24,164, an average of 3,452 per hospital. The average of 3,452 scans per hospital is lower than expected as compared to the reported utilisation in developed countries. This shows underutilisation of available capacity rather than demand limitation. Covid was a factor in 2022 and the SA National State of Emergency was only lifted on 5 April, 2022, which may have affected patient numbers. In as much as Covid could have affected patient numbers for 2022, Louw and Vangu (7) had previously raised concerns about the decline in some scans which they attributed to factors such as equipment breakdown, challenges in isotope supply and preference of some imaging modalities by referring clinicians. Clearly the reasons for the apparent stagnation of patient numbers in the SA academic hospitals need to be investigated in depth, as many other factors, such as budget constraints and staff numbers, could also play a role.

From this study carried out on South African academic hospitals, SPECT scans represented about 64%, PET scans were 30% while therapy constituted 6% of the total scans carried out in 2022. Predominance of SPECT reflects the continued reliance on conventional imaging technique. A study carried out by Sathekge et al. (10) showed under-utilisation of PET scans in some hospital departments in South Africa which was linked to limited knowledge about PET by referring physicians whereby some end up recommending use of PET at the end of the diagnostic pathway thus denying patients cost-effective treatment. There is, nevertheless, a need to carry out educational training for doctors and other healthcare practitioners on the indications of nuclear medicine scans in the patient management plan. Establishment of a patient referral system protocol could also be a key guidance to ensure deserving patients are referred accordingly (10).

There is a large inequality in access to nuclear medicine services across the globe. This has been shown by the IAEA NUMDAB which indicates that the distribution of SPECT and PET cameras varies across the different income groups of countries per million inhabitants as shown by Table 4 (8).

**Table 4:** Number of SPECT and PET scanners per million inhabitants across different countries income group.

Income group	Number of scanners per million inhabitants	
	SPECT	PET
Low-income countries	0.036	0.007
Lower middle-income countries	0.20	0.11
Upper middle-income countries	1.33	0.32
High-income countries	17.9	3.2

Source: Paez et al., 2020 (8).

In Africa, there are 0.24 SPECT scanners per million people with 85% of the total SPECT cameras in five upper-middle income countries (South Africa and North Africa). Diverseness exists in availability of PET services in Africa with seventeen cyclotrons supplying radioisotopes within the continent (4). From these South African academic hospitals study, it can be deduced that an average of 0.9 gamma cameras served an average of 2,208 patients in the hospitals while 0.9 PET-CT cameras served 1,021 patients.

For SPECT radioisotopes, ^99m^Technetium and ^131^Iodine are the most commonly used in Africa while ^18^Fluorine and ^68^Gallium are the most commonly used PET radioisotopes. ^131^Iodine, ^90^Yttrium and ^153^Samarium-153 are the most commonly used radioisotopes for radionuclide therapy in Africa (15). In this study, the most common SPECT procedures were bone scans ([^99m^Tc]Tc-MDP 23%], Lung V/Q (16%) and heart ([^99m^Tc]Tc-MIBI 12.6%]. The most commonly used PET radiopharmaceuticals were [^18^F]F-FDG (73.4%) and [^68^Ga]Ga-PSMA (17.3%)whereas for therapy were [^131^I]I-NaI (80%) and [^225^Ac]Ac-PSMA (8.8%).

Training of nuclear medicine personnel and professionals is essential in enabling nuclear medicine to attain its full potential (8). The lack of qualified staff means that some specialised tasks especially in radiopharmaceutical preparation and handling cannot be performed, hence this acts as a barrier to the provision of certain services (11). The IAEA continues to build human resource capacity in nuclear medicine which has been instrumental in the development of nuclear medicine in Africa (4). Understaffing is evident in South African nuclear medicine departments with only two hospitals having radiopharmacists (one in each). South Africa has a high number of radiopharmacy Master's graduates but there have been delays in their registration as specialists as SAPC has not allowed any radiopharmacist specialist registrations applications since 2007 with only 2 SAPC registered radiopharmacy specialists in the country (12). Many radiopharmacy graduates in South Africa are working in other fields, influenced by factors such as the availability of posts, compulsory community service, bursary repayment periods, and role substitution (12). From the study, only 2 of the responding academic hospitals indicated having a radiopharmacist each, therefore there is a need for community service places in the public sector for graduating radiopharmacists to practice and not lose their skills before they can be registered. Academic hospitals need to employ at least one radiopharmacist for their nuclear medicine departments. SAPC also needs to hasten the review of specialist applications so as to avoid delays in specialist's registration.

For a nuclear medicine department to be successful and meet its obligations and mandate, there are certain requirements that need to be met. These requirements include qualified and adequate staff, equipment and facilities that are working & serviceable, regulatory frameworks guiding the operations of the department, adequate resources needed to run the department, goodwill and support from the local governments and collaboration with various stakeholders and the private sector who offer support in various areas.

From this study, even though nuclear medicine and radiopharmacy services are well established in South Africa, there still exist gaps that need to be addressed. The various nuclear medicine departments of South African academic hospitals need to carry out a proper staffing assessment in order for them to make evidence-based requests for additional staff from the relevant authorities. By doing so, they will be are able specify their human resource needs, keeping in mind that the success of any nuclear medicine and radiopharmacy department highly depends on correct staffing (5). The full potential of nuclear medicine is realised when there is an adequate well-trained staff (13).

The study showed that the South African academic hospitals lack in-house cyclotrons. Investment in in-house hospital cyclotrons especially for hospitals that are far away from cyclotron sites should be considered as some hospitals are currently relying on supply of radiopharmaceuticals from as far as 536 kilometres on a daily basis which is expensive. By investing in in-house cyclotrons, the hospitals can also commercialise the supply of radiopharmaceuticals which will be an extra source of income hence supplementing their revenue.

There is also need to strengthen local legislation and update policies that guide radiopharmaceutical production, guidelines for licensure of radiopharmacists, registration of radiopharmaceuticals, regional pharmacopeias and involving regulatory inspectors in both hospital and radiopharmacy operations (14).

Kleynhans et al. (14), is of the opinion that a key step to ensuring the success of nuclear medicine centres is by allowing them to operate as independent departments in the hospital setting so that they have individual budgetary allocations and infrastructure incentives from the local governments. Keeping in mind that the radiopharmacy manufacturing capacity influences the success of a nuclear medicine centre, it should receive a considerable amount of the overall budget allocated (14,15).

There are basic requirements for a start-up nuclear medicine department operating SPECT-CT. They include a SPECT-CT scanner, dose calibrators, personal dosimeters and radiation monitoring devices, laminar flow biosafety cabinets, lead shielding, an appropriate location with enough space, compliance with the local and national regulations, reliable source of radiopharmaceuticals especially generators and cold kits, refrigerators for storing cold-kits, well trained staff, radiation protection measures, a good quality control program, standard operating procedures for all activities to be carried out, a system for proper waste disposal, computers and a programme for continuous education and training. Staff required will include nuclear medicine physicians, medical physicists who will also double up as the radiation protection officer, specialist nurses, radiopharmacist especially if complex specialised tasks such as cell labelling will be done, and nuclear medicine technologists/radiographers.

Clearly more work is needed to improve service levels and echo the international growth levels for Nuclear Medicine.

The limitations encountered during the conduct of these study were:

- Non-response from some participants: it had been projected at the start of data collection that data will be collected from the nine SAAHs but at the end only seven of the hospitals had given response.
- Incomplete data: some data was partially completed from the seven respondents.
- Space: space and built infrastructure was not addressed in this survey.
- Processes: this study did not address the processes in terms of Standard Operating Procedure and quality control measures.
- The effect of COVID restrictions in 2022: The National State of Emergency for COVID in the first quarter of 2022 may have skewed patient numbers,

Recommendations drawn from carrying out these study were:

- **Investing in in-house cyclotrons**

South African academic hospitals that are far away from suppliers of cyclotron produced radiopharmaceuticals are encouraged to invest in in-house cyclotrons so that they can ensure uninterrupted supply of radiopharmaceuticals as well as cut on expenses incurred from importing from other suppliers. They can also commercialise the sale of radiopharmaceuticals to supplement their revenue.

- **Staffing levels determination**

South African academic hospitals are encouraged to perform their own staff analysis on the IAEA -IRIS staff calculator with their full details of service so that they are able to identify their appropriate staff levels. Once they obtain these crucial data and by also enlisting the help of IAEA, they can advise decision makers at the national levels to employ appropriate numbers of qualified and adequate staff.

- **Registration of radiopharmacists as specialists**

Specific tasks regarding the safe use of radiopharmaceuticals should be performed by well-trained radiopharmacists which will in-turn ensure the success of a nuclear medicine centre. The government and other stakeholders should partner to avail community service places in public sectors and industry. The academic hospitals should employ at least one radiopharmacist in their nuclear medicine departments. Delays in review of radiopharmacist specialist application should also be sorted out by SAPC.

- **Continuing medical education**

Where resources are limited and to also keep the various specialist staff cadres abreast with new developments in the nuclear medicine field, short continuing courses should be introduced. The continuing medical education should also be used to train referring staff on the patient referral system to be followed and also create awareness of nuclear medicine services to other healthcare practitioners and health centers so as to promote the uptake of nuclear medicine services. Maintaining good relationships with these practitioners and health centers will also be of importance.

- **Nuclear medicine centers as independent departments**

Nuclear medicine centres could be made independent departments. By so doing, it will mean that they will benefit from having their own budgets and should also get incentives from the government and international organisation such as the IAEA. This will help them align their infrastructure to the number of patients they serve as well as the number of procedures they can perform.

- **Strengthening of regulatory framework**

There should be a speedy implementation of the existing local legislation and regulatory framework that defines how radiopharmaceutical procedures should be undertaken, that also guides the manufacture, compounding & handling of radiopharmaceuticals.

- **Standard Operating Procedures**

Facilities should be encouraged to adopt Standard Operating Procedures so as to ensure that processes and procedures are consistent and that there is quality output.

## 5 Conclusion

Data available from the study shows that nuclear medicine and radiopharmacy services are playing a critical role in patient management in South African academic hospitals especially for non-communicable diseases. Despite the availability of nuclear medicine services, their potential has not been fully utilised and heavy investments need to be made on equipment and nuclear medicine staff. The IAEA has continuously helped its member states to close these gaps through capacity building. The various governments of African states and other stakeholders are also encouraged to fully support the establishment and implementation of nuclear medicine centres and radiopharmacies through making policies that are in favour, investing in infrastructure and human resource, including medical imaging as part of national health plan, creating awareness and giving subsidies to hospitals so that medical imaging services become affordable to all citizens.

To also improve on uptake of nuclear medicine services, hospital clinicians should be encouraged to conduct regular continuing medical education to keep themselves abreast with new developments in the field and they should also embrace a multi-disciplinary approach to come up with personalised medical interventions for patients which will be beneficial to the patient as opposed to referring the patients to other colleagues for a second opinion. Proper financing models should also be adopted, which may include insurance and reimbursements to cater for the costs of these nuclear medicine procedures, thereby increasing access for the common citizen. The various nuclear medicine departments should have adequate services to meet the various training needs for future nuclear medicine and radiopharmacy staff.

The findings of the study highlight key considerations for planning radiopharmacy services which include;

- Establishment of nuclear medicine and radiopharmacy services should be demand driven based on local disease burden as opposed to adopting high income country models.
- Technetium-99 m based radiopharmaceuticals play a key role as a cost effective foundation for SPECT imaging while PET services can be introduced gradually depending on demand.
- Centralised radiopharmacy models play an important role to improve on efficiency of radiopharmaceutical supplies.
- Human resource is an integral part for the success of nuclear medicine and radiopharmacy centres.

A significant milestone that has been achieved in South Africa is the establishment of a Nuclear Medicine Research Infrastructure (NuMeRi) which is a not-for-profit company that will provide new strategic initiatives in nuclear medicine technologies and biosciences. It is a medical imaging facility dealing with drug development and clinical research. It has its headquarters at the Steve Biko Academic hospital and a NuMeRi Node for infection Imaging at the premises of Tygerberg hospital. NuMeRi will have a clinical unit comprising an 18MEV cyclotron, commercial cyclotron, 2 PET-CT, SPECT-CT, PET-MR imaging systems. It will also have a GMP radiopharmacy. The node of Infection Imaging at Tygerberg includes a PET-CT scanner and a fully equipped radiopharmacy.

The study has given a valuable insight into the pattern of utilization of nuclear medicine and radiopharmacy services in South African academic hospitals. The uptake of these services is promising and clearly demonstrates that the future of tailored and individualised treatment lies here. The existing gaps need to be addressed for effective and efficient use of these services and also substantial investments need to be done on improving the current status of nuclear medicine services and fully maximize the use of these services.

## Data Availability

The original contributions presented in the study are included in the article/supplementary material, further inquiries can be directed to the corresponding author.
